# Correction: Differential effects of aquaporin-4 channel inhibition on BOLD fMRI and diffusion fMRI responses in mouse visual cortex

**DOI:** 10.1371/journal.pone.0236380

**Published:** 2020-07-14

**Authors:** The PLOS ONE Staff

The last author's initials appear incorrectly in the citation. The publisher apologizes for the error. The correct citation is: Komaki Y, Debacker C, Djemai B, Ciobanu L, Tsurugizawa T, Le Bihan D (2020) Differential effects of aquaporin-4 channel inhibition on BOLD fMRI and diffusion fMRI responses in mouse visual cortex. PLoS ONE 15(5): e0228759. https://doi.org/10.1371/journal.pone.0228759
