# Supplementary figures and images for: Differential effects of aquaporin-4 channel inhibition on BOLD fMRI and diffusion fMRI responses in mouse visual cortex
Source: PLoS One. 2020 May 21;15(5):e0228759. doi: 10.1371/journal.pone.0228759 (PMC7241787; doi:10.1371/journal.pone.0228759)

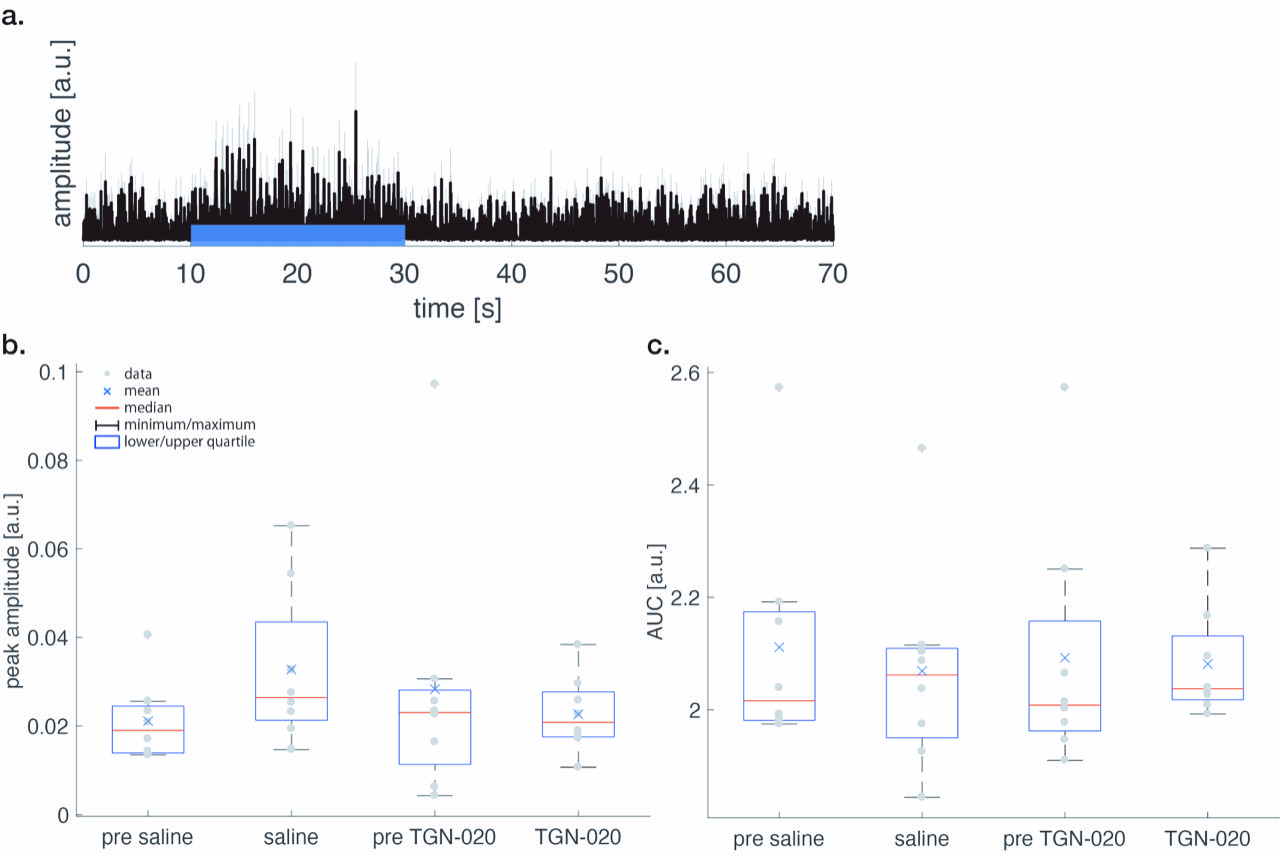

Supplement: S1 Fig — High-frequency spiking activity (a). The visual stimulus was applied between 10 and 30 seconds (a, blue block). High-frequency spiking activity obtained by applying a moving average filter of 0.001 s width window (a, black line). The peak amplitude corresponds to the maximum value during stimulation (b). The area under the curve (AUC) during the stimulation period (10–30 s) was compared with the pre-stimulation period (0–10 s) for all conditions (c). There was no significant difference in peak amplitude and AUC between the conditions (saline or TGN-020) (p<0.05 Bonferroni correction with a paired t-test.). (JPEG) [file pone.0228759.s001.jpeg]
